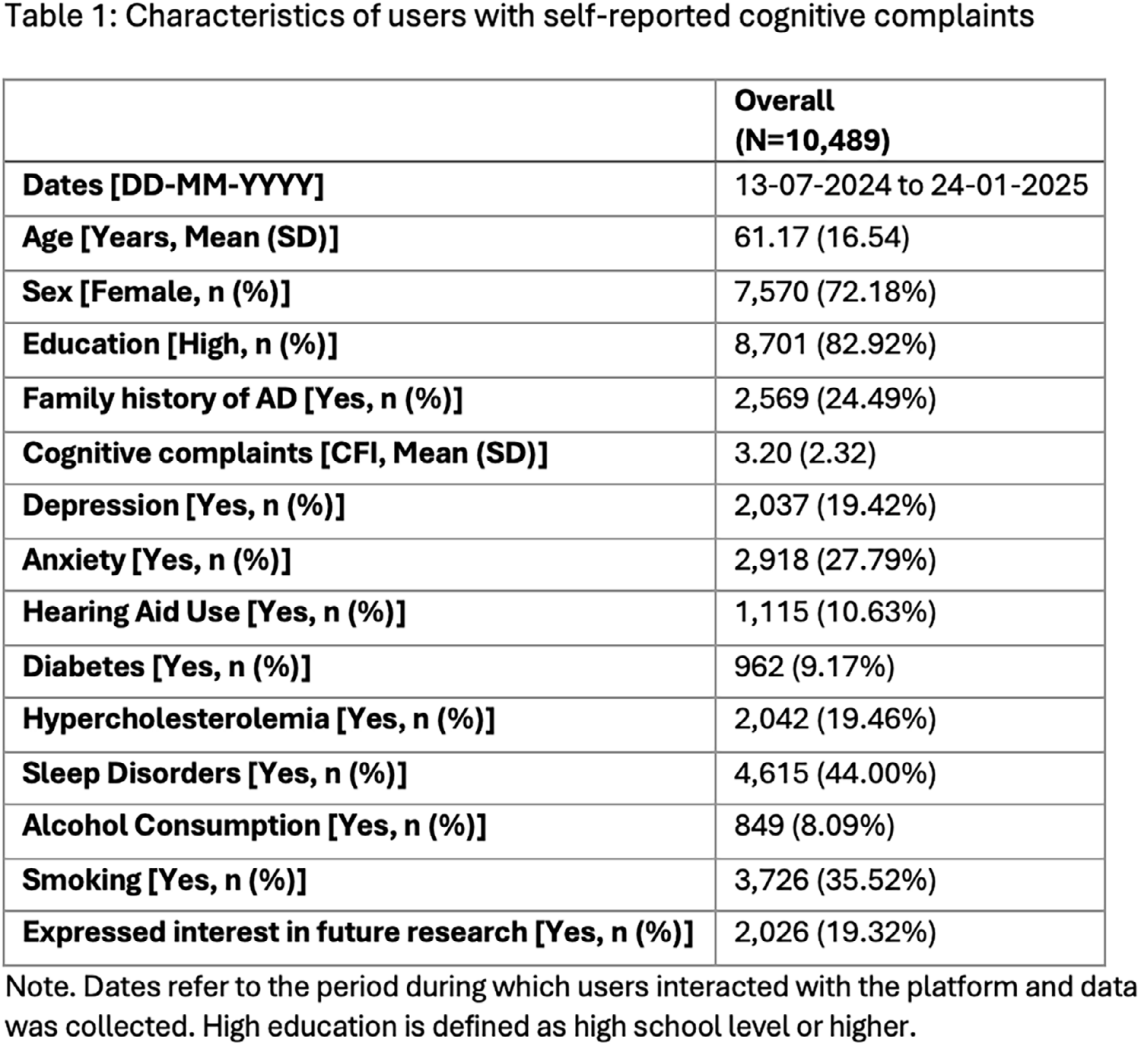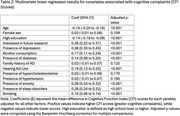# Profiling cognitive complainers using a home‐based digital platform: Insights from DocMemo users

**DOI:** 10.1002/alz70857_106802

**Published:** 2025-12-25

**Authors:** Federica Cacciamani, Graziella Mangin Laignel, Audrey Gabelle, Nicolas Beaume, Mahinthan Kengatharan, Igor Koval, Stanley Durrleman

**Affiliations:** ^1^ ARAMISLab, Sorbonne Université, Institut du Cerveau‐Paris Brain Institute‐ICM, CNRS, Inria, Inserm, AP‐HP, Hôpital de la Pitié Salpêtrière, Paris, France, France; ^2^ Qairnel, Paris, France, France; ^3^ Université de Montpellier, Montpellier, France; ^4^ Memory Resource and Research Center of Montpellier, CHU de Montpellier, Hôpital Gui de Chauliac, Montpellier, France

## Abstract

**Background:**

Understanding the profile of individuals with self‐reported cognitive complaints is essential for early identification of those at risk for Alzheimer's disease and related disorders (ADRD). Home‐based digital platforms provide unique opportunities to collect detailed data on these users and analyze factors associated with cognitive complaints. DocMemo (docmemo.fr, docmemo.com) offer cognitive screening, help schedule memory‐related visits, provide ADRD education content, and allow users to express research interest. We retrospectively analyzed DocMemo users who self‐referred for cognitive complaints to identify key factors associated with greater complaints.

**Method:**

Data was collected from users who interacted with the platform (i.e., answered questionnaires). Cognitive complaints were measured using the Cognitive Function Index (CFI), a 14‐item self‐assessment of cognitive status. The higher the score, the more intense the complaint. Multivariate linear regression was used to explain changes in CFI scores based on demographic, psychological, health‐related, and lifestyle data.

**Result:**

A total of 10,489 users were analyzed. Mean age was 61.17 years (SD: 16.54), 72.18% female, and 82.92% with a high level of education. 24.49% reported a family history of AD, and the mean CFI score was 3.20 (SD: 2.32). Other characteristics are detailed in Table 1.

Significant associations with higher CFI scores (*p* < 0.001) were found with mental health conditions, including depression (β=0.38) and anxiety (β=0.28), as well as more comorbidities, such as sleep disorders (β=0.28), hearing impairment (β=0.19) and diabetes (β=0.14). Regarding lifestyle factors, alcohol consumers (β=0.17) and smokers (β=0.10) had higher CFI scores. Individuals with higher CFI scores were more likely to express interest in being contacted for future research opportunities (β=0.26). Conversely, older individuals (β=‐0.18, *p* <0.001) and those with higher education levels (β=‐0.14, *p* <0.001) had lower CFI scores on average.

Other factors, such as sex, family history of AD, hypercholesterolemia, and hypertension were not significantly associated with CFI scores (*p* >0.05).

**Conclusion:**

DocMemo effectively identifies individuals with cognitive complaints, particularly those presenting established ADRD risk factors such as depression, sleep disorders, hearing impairment, and diabetes. These findings highlight the value of digital platforms like DocMemo in understanding the factors linked to subjective cognitive complaints and guiding targeted prevention strategies.